# Modeling the Toxicokinetics of Suspensions of Soluble Metallic Nanomaterials

**DOI:** 10.1021/acs.chemrestox.4c00177

**Published:** 2024-09-09

**Authors:** Guangchao Chen, Vivi Rottschäfer, Martina G. Vijver, Willie J. G. M. Peijnenburg

**Affiliations:** † Centre for Prevention, Lifestyle, and Health, National Institute of Public Health and the Environment (RIVM), P.O. Box 1, Bilthoven 3720 BA, The Netherlands; ‡ Mathematical Institute, Leiden University, Einsteinweg 55, Leiden 2333 CC, The Netherlands; § Korteweg-de Vries Institute for Mathematics, University of Amsterdam, P.O. Box 94248, Amsterdam 1090 GE, The Netherlands; ∥ Institute of Environmental Sciences (CML), Leiden University, Einsteinweg 2, 2333 CC, Leiden, The Netherlands; ⊥ National Institute of Public Health and the Environment (RIVM), Center for Safety of Substances and Products, P.O. Box 1, Bilthoven 3720 BA, The Netherlands

## Abstract

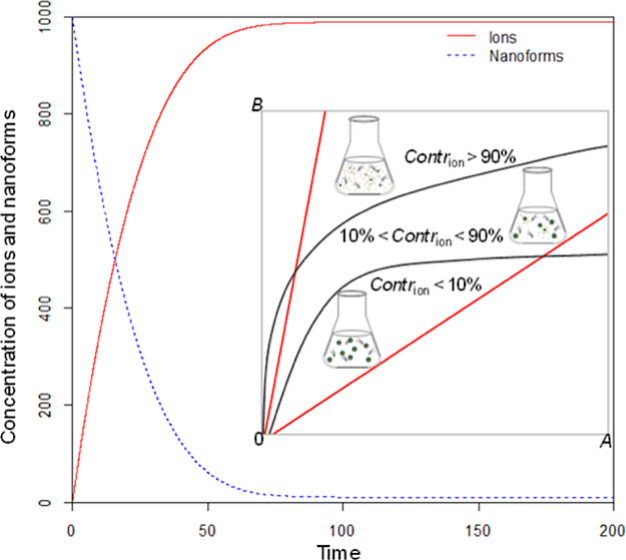

Proper risk assessment of the many new nanoforms (NFs) that are currently being developed and marketed is hindered by constraints in time and resources for testing their fate and (eco) toxicity profile. This problem has also been encountered in conventional chemical risk assessments, where the definition of related chemical groups can facilitate risk assessment for all class members. Whereas grouping and read-across methods are well established, such approaches are in the early stages of development for NFs. In this study, a modeling framework was developed for grouping NFs into distinct classes regarding the contribution of released ions to suspension-induced toxicity. The framework is based on combining dissolution rate constants of NFs with information about the toxicokinetics of the NFs and the dissolution products formed. The framework is exemplified for the specific case of suspension toxicity of metallic NFs (silver and copper). To this end, principles of mixture toxicity and dose–response modeling are integrated to derive threshold values for the key NF properties determining suspension toxicity: size, shape, and chemical composition. The threshold values thus derived offer a possible solution for the high-throughput screening of NFs according to their morphological and compositional properties in a regulatory context.

## 1 Introduction

As one of the first steps toward systematically and efficiently assessing the hazards of engineered nanomaterials (ENMs) for regulatory purposes, a framework for grouping and read-across approaches has recently been developed within the European project GRACIOUS.^1^ The approaches within the framework are tailored for nanoforms (NFs) for which similarities can be found regarding their chemical, physical, or morphological properties. For a set of NFs that are shown to be similar, it is allowed in a regulatory context to apply the information available on any of the NFs within the group to the whole set of NFs. Obviously, a proper justification for such a similarity assessment should be provided. This justification should be based on a sound hypothesis and needs to link the physicochemical properties of NFs with their hazard. The framework for grouping NFs developed by Stone et al.^1^ is based upon a set of predefined hypotheses and forms the basis for integrated approaches for testing and assessment (IATA). The IATA guides the grouping of NFs in terms of “where they go” and “what they do”. The aim of the GRACIOUS framework is to support the efficient collection of data and models that enable to support or reject each of the hypotheses.^2^

One of the predefined hypotheses within GRACIOUS, which has been integrated into an IATA by Cross et al.,^3^ deals with metallic NFs (a major part of the first generation of nanomaterials) in the aqueous environment and considers dissolution and dispersion stability as the key parameters driving the environmental fate of metallic NFs. Dissolution is an important transformation of NFs and is identified as a critical determinant of exposure in the environment.^4^ This was confirmed by the efforts within OECD with regard to the development of a Guidance Document for determining dissolution rates for nanomaterials under certain environmental conditions.^5^ After dispersion of metallic NFs in the aqueous medium, a suspension is formed in which the metallic NFs may dissolve with an NF-specific rate constant. Subsequently, a distinction can be made between nonsoluble NFs and soluble metallic NFs that release metal ions from their surface. For the latter, the adverse response of organisms to suspensions of metallic NFs can be ascribed to contributions of both particulate form (i.e., the NFs) and the ions shed from the NFs. This means that alteration of the dissolution kinetics can modify the toxic response of organisms,^6−8^ because the ratio of the concentrations of NFs and ions in the suspension will change over time. Grouping of metallic NFs thus requires assessment of:

1 NFs for which toxicity is dominated by ions, e.g., ions contribute to more than 90% of the suspension toxicity;
2 NFs for which the NFs will predominantly determine toxicity (e.g., ions contribute less than 10% to the suspension toxicity); and
3 the intermittent situation in which both ions and NFs contribute significantly to the toxicity of a suspension.

One practical significance of this classification can be interpreted as follows: in the case of group 1 (ions contribute >90%), no additional information on NF toxicity is needed for the purpose of risk assessment of an NF and information on the toxicity of the ions will suffice; in the case of group 2 (NFs contribute >90%), only information on the NF toxicity is needed; and in the case of group 3, information on the toxicity profile of both NFs and ions is needed. Overall, the release kinetics of ions and the toxic potencies of the ions and NFs will determine the time-dependent suspension toxicity. However, the challenge when aiming to quantify the toxicokinetics of suspensions of NFs lies in the fact that simple correlations between key NF properties (e.g., dissolution rate constant, density, size, and intrinsic toxicity of an NF) and ion (or NF) contribution to suspension toxicity cannot be drawn. Suspension toxicokinetics will, for instance, always be governed by exposure time and the ratio of the intrinsic toxicities of ions and NFs for soluble metallic NFs. This implies that, for example, even in the case of quickly dissolving NFs, relatively toxic NFs may contribute significantly to suspension toxicity, especially at short exposure durations. Therefore, the key question that we aim to answer here is how can we quantify the time-dependent contribution of ions and NFs to the toxicity of a suspension of an NF, explicitly considering the key properties of the NFs and the released ions and test duration?

We developed a modeling framework for assessing the toxicokinetics of suspensions of soluble NFs to answer this question. In the framework, we link the dissolution rate constant and other key NF properties and the toxicity of the released ions, to quantify the time-dependent contribution of ions and NFs to suspension toxicity. The aims of this paper are (1) deriving a dissolution-based modeling framework to predict the contribution of ions and NFs to suspension toxicity over time and (2) exemplifying the application of this framework in grouping metallic NFs into the three aforementioned classes in a regulatory context. Our results reveal how the dissolution rate constant, density, and size of NFs, together with time, jointly influence the contribution of ions and NFs in a quantitative way. To our knowledge, this is the first study that reveals the interplay of these key properties and/or factors in the toxicokinetics of soluble NFs. We focus on dissolution of spherical NFs of equal size, but the modeling framework may be extended to any other type of transformation of NFs of different morphology. The framework can also be extended to the common case of particle size distributions. For the specific case of regulatory assessment of NFs, toxicity is selected as the end point of consideration in this study. However, other end points for other applications beyond risk assessment might be selected as well. An example is provided by end points relevant for the assessment of NFs that are safe and sustainable by design.

## 2 Methods

The developed framework includes a dissolution model that calculates the concentrations of the released ions and NFs in a suspension over time. Based on this calculation, the contribution of the ions to suspension toxicity is determined. In this way, the key properties of NFs are linked to the contribution of ions and NFs to suspension toxicity, allowing for application in grouping NFs. In developing the modeling framework, we introduce the following input parameters.

- *k*: dissolution rate constant at which NFs dissolve, in g/cm^2^/h;
- ρ: density of the NF in g/cm^3^;
- *t*: time (the duration of the dissolution process) in h. At *t* = 0, the NFs are added to the medium and the dissolution process starts;
- the Hill coefficient which characterizes the shape of the concentration–response relationship of the ions is denoted by η_H^1^_ and the Hill coefficient of the NFs is given by η_H^2^_; and
- EC50_ion_ (EC50_NF_) is the effective concentration of the ions (the NFs) that induces a certain effect in 50% of the test organisms.

The developed modeling framework uses the above parameters as the model input. The outcomes of the framework are NF size as a function of time, (mass-based) particle concentration as a function of time, the ion concentration as a function of time, and the contribution of ions to suspension toxicity as a function of time.

- *D*(*t*): diameter of an NF at time *t*, in cm;
- *C*_NF_(*t*): concentration of NFs at time *t* in g/L; and
- *C*_ion_(*t*): concentration of ions at time *t* in g/L;

Details of the method are provided in Sections 2.1–2.3. The time-dependent suspension toxicity of soluble NFs is determined using the generally accepted key assumption of mixture toxicity: response addition, which can be modeled once the time–concentration profiles of the NFs and ions are obtained from the dissolution model. The response addition method^9^ was selected based on the assumption that the so-called modes of toxic action of ions and NFs are different.

An important model parameter is the Hill coefficient. In ecotoxicology (as well as in many other areas), most dose–response curves are satisfactorily described by the classical Hill equation. The Hill coefficient, *n*_H_, i.e., the steepness and shape of the dose–response curve of biota, was introduced as an empirical description by Hill.^10^ The Hill coefficient is typically used to quantify the response of a biological receptor to a stressor.^11^ As commonly prescribed in the OECD guidance document on testing of chemicals and nanomaterials,^5^ the Hill equation is a sigmoidal function that is quite often utilized to predict the dose–response relationship of NFs to a variety of different organisms and different metal-based NFs, e.g., among all to anatase and rutile *n*TiO_2_ to algae,^12,13^*n*Cu to bacteria,^14^ and *n*TiO_2_ to crustaceans.^15^

### 2.1 Model Assumptions

The assumptions made for developing the model framework are as follows:

- All NFs are of equal size and their morphology is a sphere (i.e., nanoparticles).
- Dissolution of NFs is a continuous process in the sense that the diameter of the NF decreases continuously when ions dissolve from the surface of an NF.
- A spherical NF will remain to be a sphere when ions dissolve from its surface.
- The key assumption is that the dissolution rate constant of the NF (i.e., *k*) is a characteristic of the NF. This rate constant is independent of the size of the NF but depends on the surface properties of the NF such as chemical composition and coating and on extrinsic properties like medium composition.
- At *t* = 0, the initial concentration of ions in the suspension is 0 g/L, i.e., *C*_ion_(0) = 0.
- The suspension consisting of the NFs and the released ions is homogeneous. Additional fate processes like aggregation and sedimentation are not considered.
- The NFs and ions in the suspension act via different modes of toxic action. Hence, mixture toxicity may be quantified by means of the principles of response addition.
- The EC50_NF_ in a suspension is assumed to range from EC50_ion_ to 5 × EC50_ion_ since it is commonly observed that NFs are less toxic than the corresponding metal ions.
- The Hill coefficients η_H^1^_ and η_H^2^_ are independent from the exposure duration of NFs and ions, since there is no consistent evidence that change of exposure duration would alter the shape of the concentration–response relationship of the NFs and of the ions.
- A contribution of 10 and 90% of ions to the suspension toxicity is selected as the cutoff levels for the purpose of grouping NFs. Note that these values are arbitrary choices to exemplify the developed framework. Other cutoff values can be used as well if deemed appropriate.
- Ion release reduces the driving force for dissolution, resulting in a steady-state condition in systems of finite volume (like typical in toxicity testing). In common practice, this implies establishing a steady-state concentration of ions in suspension where 100% dissolution is predicted to take place. This is assumed to derive the model. In a realistic environmental setting, conditions of finite volume will not apply.

### 2.2 Dissolution Model

The dissolution model is provided in a recent contribution from our research group.^16^ The model boils down to the following expression of the ion concentration as a function of time
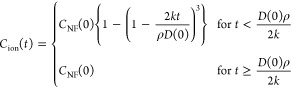
1

To simplify the above expressions, we introduce *f*(*x*) = (1 – *x*)^3^, so that . We then obtain
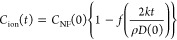
2
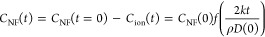
3for  and , we find that *C*_NF_(*t*) = 0.

### 2.3 Determining the Contribution of Ions to Suspension Toxicity at a Certain Time

The concentration–response relationships for the toxic effects caused by ions and NFs after a fixed period of time are given by the following expressions (a classical sigmoid dose–response model)
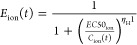
4
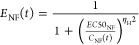
5Here, the Hill coefficient for the ions is denoted by η_H^1^_ and that of the NFs is given by η_H^2^_. EC50_ion_ (EC50_NF_) is the effective concentration of the ions (NFs) that induces a certain effect in 50% of the test organisms. Assuming that the modes of toxic action of NFs and ions are different, the response addition model can be used to derive the suspension toxicity [*E*_tox_(t)] at time *t*, based on the toxicities of ions and NFs at time *t*
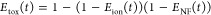
6

Finally, the contribution of ions to the suspension toxicity is given by
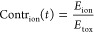
7where *E*_ion_ and *E*_tox_ are given by the above expressions. Substituting eqs 2–6 into eq 7, we obtain the expression for Contr_ion_ as a function of *t* and in terms of the aforementioned input parameters as follows
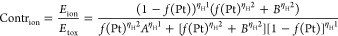
8where
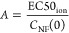
9
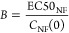
10
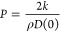
11and *C*_NF_(0) is the concentration of NFs at time 0.

The ion concentration at time 0 is assumed to be 0 mg/L. *C*_NF_(0) therefore equals the total mass concentration of the NFs. *A* and *B* are dimensionless. From expression 8, it can be seen that Contr_ion_ depends on *A*, *B*, *Pt*, η_H^1^_, and η_H^2^_; *A* and *B* are combinations of the EC50s and the initial NF concentration, and *P* is a combination of the dissolution rate constant, particle density, and the initial diameter of the NF. Equation 8 shows how these factors jointly influence Contr_ion_ and confirms that considering any of these parameters alone in classifying NFs according to Contr_ion_ would ignore the impact of any of the other factors on dissolution and suspension toxicity. Ignoring the interplay of the factors affecting the toxicokinetics of suspension toxicity would therefore be incorrect.

Even though, as indicated above, ranges for the ratio of ion toxicity vs NF toxicity were assumed, *A* and *B* can vary largely in practice, since they depend on the initial NF concentration. An important question is therefore could we still say something about Contr_ion_ based on *P* alone (at a certain time point), no matter the values of *A* and *B*? That is, whatever the EC50s of ions and NFs are (their potencies) and whatever the initial NF concentration is set to be in the experiment. For example, we could ask ourselves the question whether Contr_ion_ could always be larger than 90% for certain ranges of *P* at a certain time, regardless of the values of *A* and *B*? The answer to this question is critical for grouping NFs into the three classes based on the properties of NFs.

Inspection of eq 8 shows in addition that the Hill coefficients of the dose–response curves of ions and NFs are key determinants of the contribution of ions to suspension toxicity. We already indicated that we assume that the Hill coefficients do not change over time, whereas we could not find any information on a possible relationship between the shape of the dose–response curves for ions and NFs. We therefore assume η_H^1^_ and η_H^2^_ to be independent of each other. This implies that, in principle, there are an infinite number of combinations of η_H^1^_ and η_H^2^_ that can be used in the exemplification of the relationship between Contr_ion_ and *P*. To illustrate the interplay between P and ion contributions, we provide a few illustrations in Section 2.4. The examples are based on realistic values for the density of the particles while assuming a limited set of values of the Hill coefficients.

**Figure 1:**
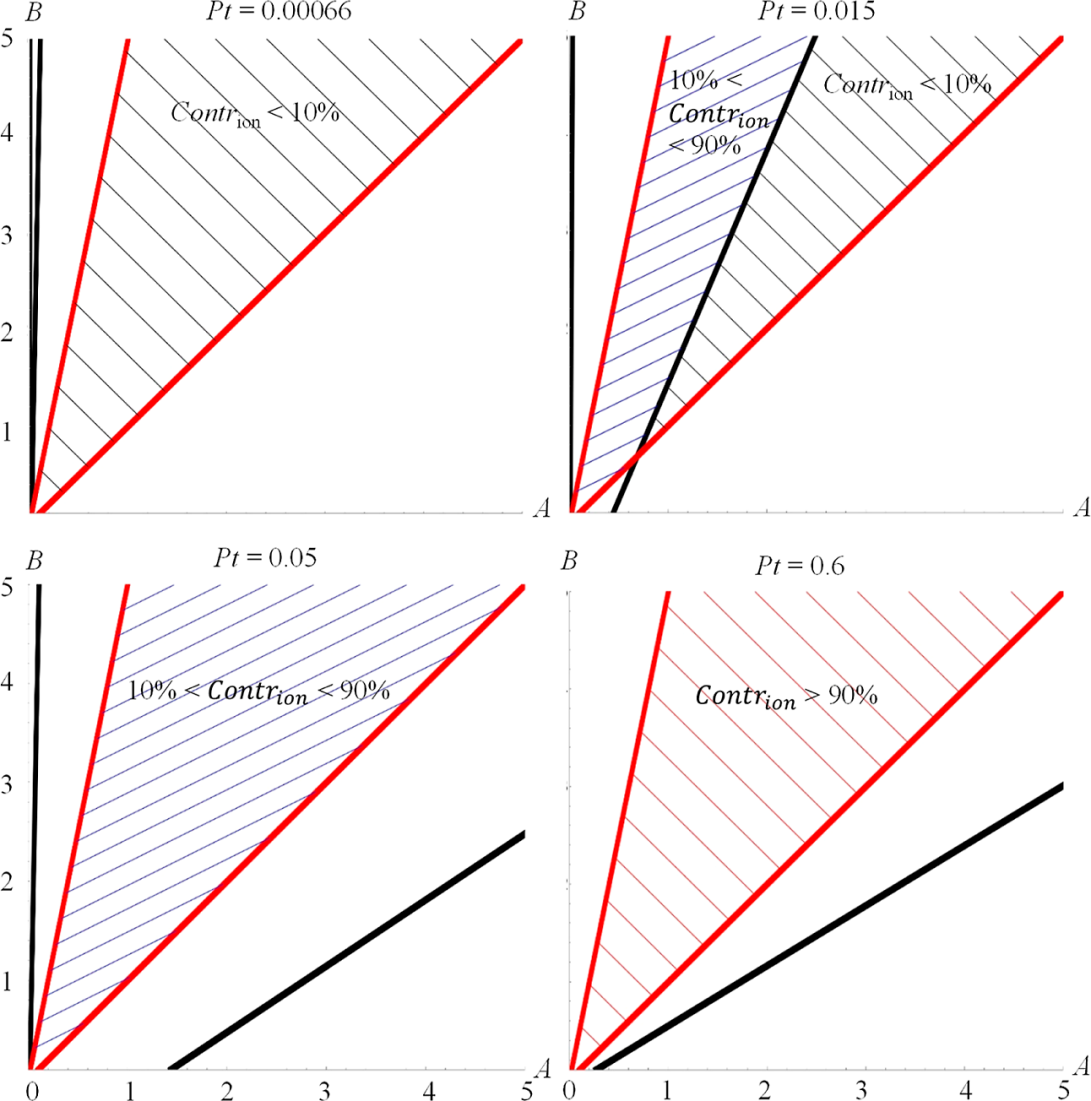
Illustration of the possible situations of Contr_ion_ < 10% (shadowed by gray lines as in top left), 10% < Contr_ion_ < 90% (shadowed by blue lines as in top right and bottom left), and/or 90% < Contr_ion_ (shadowed by brown lines as in bottom right) when *Pt* takes different values, for the scenario η_H^1^_ = η_H^2^_ = 1. The boundaries between these areas are marked by black lines. The lines on which *A* = *B* and *B* = 5*A* are given in red; the area between *A* = *B* and *B* = 5*A* is shadowed by the gray, blue, and/or brown lines as described above, which indicates the reasonable range based on the assumptions. Top left, for Pt = 0.00067; here, always Contr_ion_ < 10%. Top right: *Pt* = 0.015, there are 2 possibilities: a region where Contr_ion_ < 10% and a region where 10% < Contr_ion_ < 90%. Bottom left: *Pt* = 0.05, there is 1 region: 10% < Contr_ion_ < 90%. Bottom right: *Pt* = 0.6; in this case, Contr_ion_ > 90% always.

**Figure 2:**
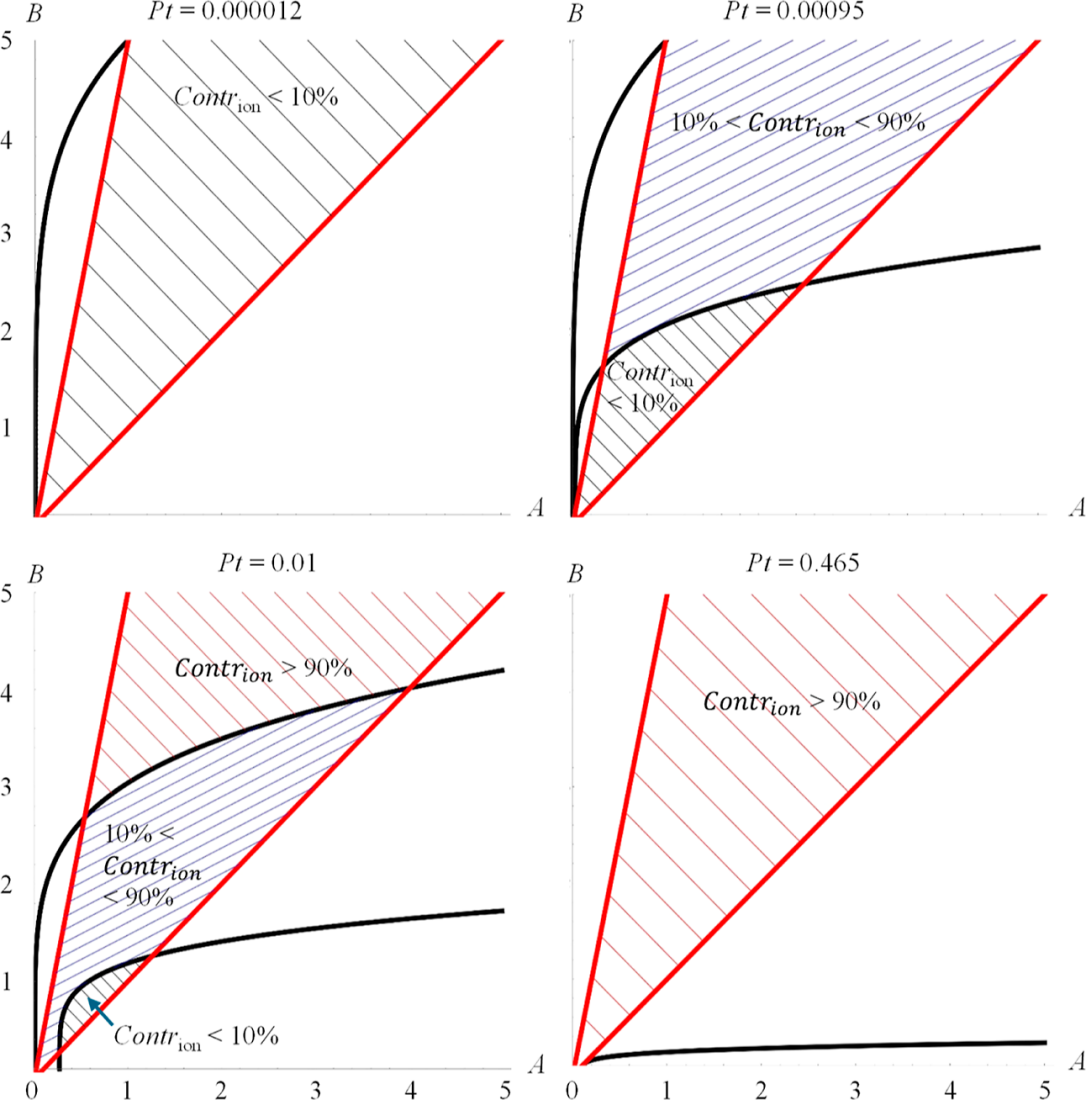
Illustration of the possible situations of Contr_ion_ < 10% (shadowed by gray lines as in top left), 10% < Contr_ion_ < 90% (shadowed by blue lines as in top right and bottom left), and/or 90% < Contr_ion_ (shadowed by brown lines as in bottom right) when *Pt* takes different values, for the scenario η_H^1^_ = 1 and η_H^2^_ = 5. The boundaries between these areas are marked by black lines. The lines on which *A* = *B* and *B* = 5*A* are given in red; the area between *A* = *B* and *B* = 5*A* is shadowed by the gray, blue, and/or brown lines as described above, which indicates the reasonable range based on the assumptions. Top left, for *Pt* = 0.000012; here, Contr_ion_ < 0.1 always. Top right: *Pt* = 0.00095, there are 2 possibilities: a region where Contr_ion_ < 10% and a region where 10% < Contr_ion_ < 90%. Bottom left: *Pt* = 0.01, there are 3 regions: either Contr_ion_ < 10%, 10% < Contr_ion_ < 90%, or Contr_ion_ > 90%. Bottom right: Pt = 0.465; in this case, Contr_ion_ > 90% always.

The examples are also illustrated with figures (Figure 1 and 2) for different scenarios. In each figure, two red lines are drawn to indicate the area between *A* = *B* and *B* = 5*A*. The area between *A* = *B* and *B* = 5*A* is shadowed which indicates the reasonable range to be examined based on the assumptions. Meanwhile, black lines are drawn to indicate the boundaries between the areas where the contribution of the ion formed is either <10%, or in between 10 and 90%, or >90%. The area for Contr_ion_ < 10% is shadowed by gray lines, the area representing the case where 10% < Contr_ion_ < 90% is shadowed by blues lines, and brown lines are used for the case of Contr_ion_ > 90%.

### 2.4 Example Scenarios

The link between *P* and Contr_ion_ was investigated based on two key settings.

First, various combinations of η_H^1^_ and η_H^2^_ were chosen to represent different scenarios with regard to the toxic potencies of the ions and NFs. For illustrative purposes, three scenarios were selected: η_H^1^_ = η_H^2^_ = 1; η_H^1^_ = 1, η_H^2^_ = 5; and η_H^1^_ = 4, η_H^2^_ = 8, thus covering a range of values of the Hill coefficients and a ratio of η_H^1^_ and η_H^2^_ that ranges by a factor of 8.

Second, regarding the values of *A* and *B*, we already indicated that particles are assumed to be up to 5 times less toxic than metal ions. This implies that *A* < *B* < 5*A*. We furthermore considered that it is common practice in toxicity testing to perform a range finding test after which a limited number of test concentrations are selected for the actual toxicity testing. We assume these concentrations to range from 0.1 to 5 times the EC50_NF_. This implies that 0.1 < *B* < 5.

Application of the model equations given above learned that there indeed exist ranges for Pt in which Contr_ion_ < 10% while satisfying the condition of *A* ≤ *B* ≤ 5*A*, i.e., contribution of the ions is always less than 10%. Similarly, ranges for Pt in which the contribution of the ions is in between 10 and 90% and above 90% can be identified. To translate these ranges of Pt into practice for the purpose of grouping NFs into the three classes, the following cases for the NFs are chosen to exemplify the interpretation of the model outcomes for each scenario:

- As an example, we selected the cases of the often-studied silver and copper NFs, with density ρ = 10.49 and 8.95 g/cm^3^, respectively.
- We selected three commonly applied test durations: *t* = 72, 168, and 672 h.
- The diameter of the NFs [*D*(0)] was assumed to be 10 and 100 nm at *t* = 0.

## 3 Results

In Sections 3.1–3.3, we describe for each scenario the ranges of Pt that lead to different classifications of the NFs. For this purpose, we plot the values of *B* (eq 10) as a function of *A* (eq 9). In Section 3.4, we use actual examples to show how to translate these ranges of Pt into different cases for NFs.

### 3.1 Scenario η_H^1^_ = η_H^2^_ = 1

The first selected scenario is when the ions and NFs have the same value of the Hill coefficient of the concentration–response relationship, i.e., η_H^1^_ = η_H^2^_ = 1. In this case, the following ranges of Pt were obtained, each of which leads to different possibilities for Contr_ion_ (also see Figure 1):

- For Pt < 0.00067, we find that Contr_ion_ < 10% always (Figure 1 top left).
- For 0.00067 < Pt < 0.0295, there are 2 possibilities: there exists a region where Contr_ion_ < 10% and a region where 10% < Contr_ion_ < 90% (Figure 1 top right).
- For 0.0295 < Pt < 0.103, we find that always 10% < Contr_ion_ < 90% (Figure 1 bottom left).
- When 0.103 < Pt < 0.521, there are 2 possibilities: there exists a region where 10% < Contr_ion_ < 90% and a region where Contr_ion_ > 90%.
- For Pt > 0.521, we find that Contr_ion_ > 90% (Figure 1 bottom right).

From these results, we conclude that in this scenario, the NFs can be grouped into the first class when Pt > 0.521: NFs for which toxicity is dominated by the ions formed. When Pt < 0.00067, an NF can be classified into the second class; and for 0.0295 < *Pt* < 0.103, both ions and NFs contribute significantly to the suspension toxicity (i.e., the third class). In Section 3.4, we illustrate how these ranges of *Pt* can be translated to concise statements for NFs. It should be noted that in this specific scenario, the cutoff value of *Pt* of 0.00067 is not a realistic value in terms of typical particle properties.

### 3.2 Scenario η_H^1^_ = 1 and η_H^2^_ = 5

In the second scenario, we analyze the case that the dose–response curve for the ions is steeper than the dose–response curve for the NFs, with η_H^1^_ = 1 and η_H^2^_ = 5. This results in (see also Figure 2):

- For Pt < 0.000012, we find that Contr_ion_ < 10% always (see Figure 2 top left).
- When Pt takes the range from 0.000012 < Pt < 0.00095, there are 2 possibilities: there exists a region where Contr_ion_ < 10% and a region where 10% < Contr_ion_ < 90% (see Figure 2 top right).
- For 0.00095 < Pt < 0.022, we find that there are 3 regions: either Contr_ion_ < 10%, 10% < Contr_ion_ < 90%, or Contr_ion_ > 90% (Figure 2 bottom left).
- When 0.022 < Pt < 0.465, there are 2 possibilities: there exists a region where 10% < Contr_ion_ < 90% and a region where Contr_ion_ > 90%.
- For Pt > 0.465, we find that Contr_ion_ > 90% (see Figure 2, bottom right).

### 3.3 Scenario η_H^1^_ = 4 and η_H^2^_ = 8

The last selected scenarios are η_H^1^_ = 4 and η_H^2^_ = 8, which represents the situation when the dose–response curve of the ions is twice the slope of the dose–response curve of the NF. The predicted ranges of Pt are as follows:

- For Pt < 0.0074, we find that Contr_ion_ < 10%.
- When 0.0074< Pt < 0.02, there are 2 possibilities: there exists a region where Contr_ion_ < 10% and a region where 10% < Contr_ion_ < 90%.
- For 0.02 < *Pt* < 0.124, we find that there are 3 regions: either Contr_ion_ < 10%, 10% < Contr_ion_< 90%, or Contr_ion_ > 90%.
- When 0.124 < *Pt* < 0.26, there are 2 possibilities: there exists a region where 10% < Contr_ion_ < 90% and a region where Contr_ion_ > 90%.
- For *Pt* > 0.26, we find that Contr_ion_ > 90% always.

### 3.4 Example Illustrations

In Sections 3.1–3.3, we obtained various choices of *Pt* for which Contr_ion_ falls either in the range of <10, or 10–90%, or >90%, i.e., either of the three aforementioned classes that an NF can be grouped into. Here, we give examples of how to interpret these ranges of *Pt* in terms of actual cases of NFs. We take silver and copper NFs as examples (density 10.49 and 8.95 g/cm3, respectively). These metallic nanoparticles were selected as they constitute some of the most studied nanomaterials, especially regarding fate and (aquatic) effect assessment of NFs.^16−18^ First, we study a silver NF with a diameter of 10 nm for which we want to evaluate the suspension toxicity after 72 h of dissolution. Moreover, we assume that the Hill coefficients for the ions and NFs are both 1 (η_H^1^_ = η_H^2^_ = 1). For this specific scenario, we found in Section 3.1 that when *Pt* > 0.521, Contr_ion_ will always be larger than 90%. Therefore, based on expression (11), we assume that
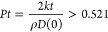

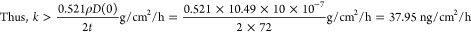


where in the second step, we write the diameter in cm, and in the last step, we transform from g/cm^2^ h to ng/cm^2^ h. This means that if the measured dissolution rate constant of this silver NF is larger than 37.95 ng/cm^2^/h, then after 72 h of exposure, the released ions contribute more than 90% to the suspension toxicity. In this case, it would be sufficient to use the information on the toxicity of silver ions for evaluating the hazard of this NF.

Vice versa, the information on the boundaries of *Pt* can also be applied to the situation in which the dissolution rate constant is already measured. Let us assume the measured dissolution rate constant to be half of the critical value for the case of 72 h of exposure (i.e., 18.98 ng/cm^2^/h), and one would be considering how long the dissolution would take until the suspension toxicity is caused predominantly by the ions:

Based on expression (11), we again have in this scenario
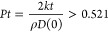


Therefore, *t* >  h = 144 h. This implies that if the dissolution takes place for more than 144 h, the suspension toxicity is mainly caused by released ions. In the same way, we could also obtain the ranges for the density and diameter of an NF given that the values of all other parameters for this NF are known.

Tables 1 and 2 illustrate some scenarios for the silver and copper NFs, with different exposure times (duration of dissolution), Hill coefficients, and NF sizes. On the basis of the described thresholds of *Pt* as deduced in paragraphs 3.1, 3.2, and 3.3, different ranges for *k* were calculated. This was done for the cases when Contr_ion_ < 10% always or Contr_ion_ > 90% always. We take a few examples from Tables 1 and 2 to explain how to interpret these values.

**Table 1:** Prediction Results for Silver NFs (Density 10.49 g/cm^3^) in Terms of Critical Values for *Pt* and *k*

Scenario	time (h)	*D* (nm)	upper bound of *Pt* for which Contr_ion <10%	lower bound of *Pt* for which Contr_ion >90%	upper bound of *k* (ng/cm^2^/h) for which Contr_ion_ < 10%	lower bound of *k* (ng/cm^2^/h) for which Contr_ion_ > 90%
η_H__^1^_= η_H__^2^_= 1	72	10	0.00067	0.521	0.05^a^	37.95
	168	10	0.00067	0.521	0.02^a^	16.27
	672	10	0.00067	0.521	0.01^a^	4.07
	72	100	0.00067	0.521	0.49	379.53
	168	100	0.00067	0.521	0.21	162.66
	672	100	0.00067	0.521	0.05^a^	40.66
η_H__^1^_= 1, η_H__^2^_= 5	72	10	0.000012	0.465	0.0009^a^	33.87
	168	10	0.000012	0.465	0.0004^a^	14.52
	672	10	0.000012	0.465	0.0001^a^	3.63
	72	100	0.000012	0.465	0.01^a^	338.74
	168	100	0.000012	0.465	0.0037^a^	145.17
	672	100	0.000012	0.465	0.0009^a^	36.29
η_H__^1^_= 4, η_H__^2^_= 8	72	10	0.0074	0.26	0.54	18.94
	168	10	0.0074	0.26	0.23	8.12
	672	10	0.0074	0.26	0.06	2.03
	72	100	0.0074	0.26	5.39	189.40
	168	100	0.0074	0.26	2.31	81.17
	672	100	0.0074	0.26	0.58	20.29

a Model prediction, but these values might be unrealistic (*k* ≤ 0.01 ng/cm^2^/h) in the sense that they cannot be determined experimentally.

**Table 2:** Prediction Results for Copper NFs (Density 8.95 g/cm^3^) in Terms of Critical Values for Pt and *k*

scenario	time (h)	*D* (nm)	upper bound of *Pt* for which Contr_ion < 10%	lower bound of *Pt* for which Contr_ion > 90%	upper bound of *k* (ng/cm^2^/h) for which Contr_ion_ < 10%	lower bound of *k* (ng/cm^2^/h) for which Contr_ion_ > 90%
η_H__^1^_= η_H__^2^_= 1	72	10	0.00067	0.521	0.04	32.35
	168	10	0.00067	0.521	0.02	13.86
	672	10	0.00067	0.521	0.0045^a^	3.47
	72	100	0.00067	0.521	0.42	323.45
	168	100	0.00067	0.521	0.18	138.62
	672	100	0.00067	0.521	0.04	34.66
η_H__^1^_= 1, η_H__^1^_= 5	72	10	0.000012	0.465	0.0007^a^	28.87
	168	10	0.000012	0.465	0.0003^a^	12.37
	672	10	0.000012	0.465	0.0001^a^	3.09
	72	100	0.000012	0.465	0.01^a^	288.69
	168	100	0.000012	0.465	0.0032^a^	123.72
	672	100	0.000012	0.465	0.0008^a^	30.93
η_H__^1^_= 4, η_H__^2^_= 8	72	10	0.0074	0.26	0.46	16.14
	168	10	0.0074	0.26	0.20	6.92
	672	10	0.0074	0.26	0.05	1.73
	72	100	0.0074	0.26	4.59	161.42
	168	100	0.0074	0.26	1.97	69.18
	672	100	0.0074	0.26	0.49	17.29

a Model prediction, but these values might be unrealistic (*k* ≤ 0.01 ng/cm^2^/h) in the sense that they cannot be determined experimentally.

It can for instance be derived from Table 1 that for a silver NF of 10 nm diameter for which η_H^1^_ = 1 and η_H^2^_ = 5, and for which the measured value of *k* is less than 0.0004 ng/cm^2^/h, the silver ions will contribute for less than 10% to the toxicity of a suspension of this NF after 168 h of dissolution. However, when *k* is measured to be at least 14.52 ng/cm^2^/h, the suspension toxicity will mostly be caused by the ions (Contr_ion_ > 90%). For a copper NF of 100 nm diameter with η_H^1^_ = 4 and η_H^2^_ = 8, after 72 h of exposure, the Contr_ion_ will be <10% if *k* is less than 4.59 ng/cm^2^/h (Table 2). When *k* > 161.42 ng/cm^2^/h, ions are by far the major cause of suspension toxicity (Contr_ion_ > 90%).

Similar to the examples provided in Tables 1 and 2, users could also work out other cases for the dissolution constant *k* (given that the values of the other parameters are known); or for other parameters other than *k* such as dissolution time or density, as shown above.

## 4 Discussion and Conclusions

Grouping and read across/analogue approaches have already been established for many years for chemical substances to meet regulatory data requirements.^19−21^ The main aim of these alternative approaches is to facilitate efficient risk assessment, among others by allowing one to deviate from performing standard test requirements that are costly and commonly require test animals. The aim of these approaches is to predict the toxicological (or physical–chemical or fate-related) profiles of chemical substances based on structural properties and similarities while avoiding tedious and costly laboratory testing which often also involves the use of test animals. This study presents the basis of a modeling framework in which the key properties of metallic NFs are linked to the toxicological profiles of their suspensions: the time-dependent contribution of the released ions to suspension toxicity. This linkage is of great interest to classify NFs into different groups (whether or not ions are the dominant contribution to suspension toxicity) according to the key NF properties affecting dissolution and toxicity, such as size, dissolution rate constant, and density. An important finding of our study is how these three properties (together with time) jointly influence the contribution of ions in a quantitative way. This approach is intended to reduce the amount of testing necessary to determine the effects of all NFs of a group (e.g., spherical silver NFs) and to offer a possible way of high-throughput screening in classifying NFs for the purpose of risk assessment.

This study also evidenced that grouping and read across of NFs based on merely molecular structural similarity (i.e., considering only the type of NFs) are not feasible for an adequate hazard assessment while avoiding individual testing of a large number of different NFs. In that context, key physicochemical properties such as morphology (e.g., size and shape), composition (e.g., density and surface properties), and fate descriptors like the dissolution rate in relevant media are indispensable. This is a distinct difference compared to the grouping and read across of soluble substances for which structural similarities and structurally similar products of the physical or biological degradation processes are important references in the classification scheme as for instance developed and applied within REACH.^22^ It is to be noted in this respect that the modeling framework allows us to incorporate the impact of variations in environmental conditions and variations in the composition of exposure media used for dissolution and toxicity testing in the time-dependent modeling of the toxicity of suspensions of soluble NFs. The impact of varying the conditions is primarily reflected in the value of the dissolution rate constant. This rate constant is dependent on the intrinsic properties of NF and on the extrinsic properties of the medium. pH is, for instance, an important parameter in this respect for the specific case of metallic NFs, with decreasing pH commonly increasing the dissolution rate constant.

Some limitations also need to be addressed. To develop a modeling framework, various simplifications and assumptions were made in the study. For instance, the NFs considered are assumed to be only spherical and of equal size (no size distribution). The mathematical inclusion of size distributions is, however, well feasible, and this is an issue for the further improvement of the framework. Another key simplification is that the dissolution rate constant at the surface of an NF is a characteristic of the NF and is thus independent of the particle size. Smaller particles of an NF may, however, possess a surface reactivity higher than that of larger particles. An issue related to this phenomenon is the fact that the framework is applicable to particles of different morphology. However, different from spherical particles of different sizes, the reactivities of particles of different morphology might be different even in the case of particles with the same chemical composition. For instance, platelet transformation occurs preferentially at the edges of platelets. This implies that the dissolution rate constant is size dependent when considering the platelets as a whole. In this specific case, one option is to assume the dissolution rate to be constant only for the ions being shed at the edges of the particle. Obviously, experimental data are needed to validate these kinds of assumptions.

Thereupon, it is to be realized that NFs are commonly present in aqueous suspensions as aggregates of varying size, while in some cases, even sedimentation occurs during dissolution and/or toxicity testing. It is likely that modulation of aggregation as induced by the composition of the exposure medium affects the actual rate of dissolution of NFs of similar composition and size. Aggregation will reduce the effective particle surface available for interaction with the medium and is thus expected to reduce the effective dissolution rate constant. Unfortunately, no kinetic data are currently available to quantify the impact of aggregation and sedimentation on the NF dissolution.

In the framework, we made assumptions on the potencies of NFs and their ions: the EC50_NF_ was assumed to range from EC50_ion_ to 5*EC50_ion_. Thereupon, the Hill coefficients of the NFs and ions were also assumed to be known. Information about typical values of the Hill coefficients is critical to model the time-dependent toxicokinetics of NFs. This information may be derived and enriched by analyzing large sets of concentration–response data of NFs of different morphologies, chemical compositions, and shape. The information thus gained can be used to improve the settings of the framework by selecting common experimental values for the key parameters *A*, *B*, and *Pt*. An example of a study in which Hill coefficients were derived based on a large set of dose–response data for metallic NFs is provided by Bunmahotama et al.^23^ It is finally to be noted that in this study, the cutoff limits of 10 and 90% for the contribution of ions to suspension toxicity are chosen for illustration purposes. When deemed valid, other thresholds (e.g., 1 or 5%, and 99 or 95%) could also be used for the classification.

To conclude:

- This study presents a modeling framework for grouping NFs into three classes regarding their contribution to suspension toxicity (ions contribute <10, 10–90, and >90%).
- The most important NF properties determining the contribution of NFs to suspension toxicity are the size of the NF, dissolution rate constant, and density.
- Exposure time (or duration of dissolution) is also a key factor that determines the boundary values for the key descriptors that take a central role in the modeling framework. The implication of this finding is that grouping of NFs does not only depend on the end point of toxicity but also on the duration of the toxicity test that is modeled.
- The Hill coefficients of the dose–response curves for NFs and ions play an important role as well in determining the contribution of ions to suspension toxicity.
- This approach offers a possible solution for high-throughput screening of NFs according to their morphological and compositional properties in a regulatory context.
